# Plasma Clusterin and the CLU Gene rs11136000 Variant Are Associated with Mild Cognitive Impairment in Type 2 Diabetic Patients

**DOI:** 10.3389/fnagi.2016.00179

**Published:** 2016-07-28

**Authors:** Rongrong Cai, Jing Han, Jie Sun, Rong Huang, Sai Tian, Yanjue Shen, Xue Dong, Wenqing Xia, Shaohua Wang

**Affiliations:** ^1^Department of Endocrinology, The Affiliated ZhongDa Hospital of Southeast UniversityNanjing, China; ^2^Medical School of Southeast UniversityNanjing, China

**Keywords:** clusterin, mild cognitive impairment, polymorphism, type 2 diabetes mellitus, memory

## Abstract

**Objective:** Type 2 diabetes mellitus (T2DM) is related to an elevated risk of mild cognitive impairment (MCI). Plasma clusterin is reported associated with the early pathology of Alzheimer's disease (AD) and longitudinal brain atrophy in subjects with MCI. The rs11136000 single nucleotide polymorphism within the clusterin (CLU) gene is also associated with the risk of AD. We aimed to investigate the associations among plasma clusterin, rs11136000 genotype and T2DM-associated MCI.

**Methods:** A total of 231 T2DM patients, including 126 MCI and 105 cognitively healthy controls were enrolled in this study. Demographic parameters were collected and neuropsychological tests were conducted. Plasma clusterin and CLU rs11136000 genotype were examined.

**Results:** Plasma clusterin was significantly higher in MCI patients than in control group (*p* = 0.007). In subjects with MCI, plasma clusterin level was negatively correlated with Montreal cognitive assessment and auditory verbal learning test_delayed recall scores (*p* = 0.027 and *p* = 0.020, respectively). After adjustment for age, educational attainment, and gender, carriers of rs11136000 TT genotype demonstrated reduced risk for MCI compared with the CC genotype carriers (*OR* = 0.158, χ^2^ = 4.113, *p* = 0.043). Multivariable regression model showed that educational attainment, duration of diabetes, high-density lipoprotein cholesterol (HDL-c), and plasma clusterin levels are associated with MCI in T2DM patients.

**Conclusions:** Plasma clusterin was associated with MCI and may reflect a protective response in T2DM patients. TT genotype exhibited a reduced risk of MCI compared to CC genotype. Further investigations should be conducted to determine the role of clusterin in cognitive decline.

**Trial registration** Advanced Glycation End Products Induced Cognitive Impairment in Diabetes: BDNF Signal Meditated Hippocampal Neurogenesis ChiCTR-OCC-15006060; http://www.chictr.org.cn/showproj.aspx?proj=10536

## Introduction

With the development of living condition and the increased number of aging population, type 2 diabetes mellitus (T2DM) is becoming prevalent. According to International Diabetes Federation atlas, the prevalence of T2DM were 8.8 and 10.6% in the world and China by 2015, respectively (Available)^1^. Diabetes itself and its complications seriously threaten human health; diabetes-induced lesions in the central nervous system has increasingly received attention (Van Harten et al., 2006). Studies have suggested that T2DM patients suffer from mild cognitive impairment (MCI) (Cukierman et al., 2005; Cheng et al., 2012), a transitional stage between normal cognitive aging and dementia (Mariani et al., 2007). A review combined 42 studies showed that the prevalence of MCI was 3–42% and the incidence of MCI was 21.5–71.3 per 1000 person-years, respectively (Ward et al., 2012). The etiology of diabetes-related cognitive impairment is complicated and associated with many factors, such as insulin impairment, high oxidative stress, mitochondrial dysfunction, and dyslipidemia (Ahmad, 2013). However, the underlying mechanisms remain unclear.

Clusterin, also named apolipoprotein J, is a multifunctional lipoprotein and exists at high levels in the brain, plasma, liver, and cerebrospinal fluid (Mullan et al., 2013). This stress-induced protein is associated with a sort of physiological functions, including apoptosis, lipid transport, endocrine secretion, complement regulation, membrane protection, sperm maturation, and promotion of cell interactions, as well as serves as complement inhibitor and molecular chaperone (Jones and Jomary, 2002; Nuutinen et al., 2009). Studies have shown that clusterin, similar to its predecessor apolipoprotein E (ApoE), plays a vital role in the pathogenesis of Alzheimer's disease (AD) (Sihlbom et al., 2008; Yu and Tan, 2012). In addition, plasma clusterin is related to brain atrophy, baseline disease severity, and rapid clinical progression in AD (Thambisetty et al., 2010). A *vitro* study has shown that clusterin influences amyloid-beta (Aβ) clearance (Holtzman, 2004), and enhances Aβ uptake by adult human astrocytes (Nielsen et al., 2010). Moreover, clusterin is associated with the early stages of AD pathology (Lidstrom et al., 1998), and plasma clusterin is related to longitudinal brain atrophy in MCI patients (Thambisetty et al., 2012). In T2DM, a significantly increased level of plasma clusterin was found (Trougakos et al., 2002) and clusterin might be an useful biomarker for detecting the early stage of diabetic retinopathy (Jin et al., 2016). Taken together, we hypothesize that plasma clusterin is related with MCI in T2DM patients.

CLU gene is located on chromosome 8p21 and contains 9 exons. It is suggested that CLU gene is involved in many severe physiological disease such as diabetes and neuron degeneration (Trougakos and Gonos, 2006; Meerzaman et al., 2014; Park et al., 2014). What's more, CLU is associated with intensified the deleterious effects of T2DM on neurocognitive slowing (McFall et al., 2015). Recent genome-wide association studies have reported that the single nucleotide polymorphism (SNP) rs11136000 in the CLU gene is associated with late-onset AD (LOAD) among Caucasians (Harold et al., 2009; Lambert et al., 2009). A reduced risk of LOAD in individuals with TT genotype than CC genotype was found. CC genotype carriers have hyperactivation in hippocampus, frontal cortex, and posterior cingulate cortex compared to TT carriers when performing working memory task (Ma et al., 2011; Lancaster et al., 2015). In addition, subjects carried the CC allele exhibited the highest Aβ deposition than TC and TT allele carriers (Tan et al., 2016). Consistently, T allele of rs1113600 in CLU gene is associated with an obviously reduced risk of AD development (Harold et al., 2009; Lambert et al., 2009), and the C allele expressed a 1.16 greater odds of AD than T allele (Bertram et al., 2007). However, conflicting results were obtained from Chinese population. Chen et al have shown that rs11136000 polymorphism is associated with AD (Chen et al., 2012). By contrast, two studies have found that rs11136000 is either weakly associated or not associated with AD (Yu et al., 2010; Ma et al., 2011). In addition, no study has investigated the association of CLU rs11136000 polymorphism with diabetes-related MCI.

From the above, potential roles for both clusterin protein concentration and CLU gene exist in cognitive impairment pathological process. One possible mechanism for the relationship may be variant of CLU modifiers of plasma clusterin expression. A previous study identified 11% higher level of plasma clusterin in rs11136000 TT carriers than CC carriers in cognitive healthy individuals (Schurmann et al., 2011). By contrast, a recent published study found that TT homozygotes had lower plasma clusterin level compared to CC homozygotes in subjects with healthy-cognition (Mullan et al., 2013). Considering the conflicting results and deficient data in MCI patients, we also explore whether plasma clusterin concentration is influenced by SNP rs11136000 in CLU gene.

This study aimed to explore the association of plasma clusterin with cognitive performances, and investigate whether SNP rs11136000 in CLU gene is related with plasma clusterin expression and T2DM-associated MCI.

## Materials and methods

### Study population

This study was conducted among 231 T2DM patients who were hospitalized in the Department of Endocrinology at the Affiliated Zhongda Hospital of Southeast University. The participants were all Chinese Han, and they provided written informed consent which is approved by the Research Ethics Committee of the Affiliated Zhongda Hospital of Southeast University.

We recruited 40–80-year-old patients who can understand and cooperate with the study procedures. All patients were diagnosed with T2DM based on the 1999 World Health Organization criteria (Alberti and Zimmet, 1998). Among the participants, 126 were diabetic patients with MCI and 105 were diabetic patients showing healthy cognition. All MCI patients satisfied the diagnostic criteria proposed by the MCI Working Group of the European Consortium on AD, as follows: (1) cognitive complaints from patients or their families; (2) decline in cognitive function in the past year relative to previous abilities, as reported by a patient or by an informant (clinical dementia rating (CDR) score of 0.5); (3) cognitive disorders as evidenced by clinical evaluation (impairment in memory or in other cognitive domain); (4) absence of major limitations in activities of daily living (ADL) score of 26; and (5) absence of dementia (Portet et al., 2006). The exclusion criteria were as follows: severe hypoglycemia, hyperosmolar non-ketotic diabetic coma, diabetic ketoacidosis, or other acute diabetic complications; history of stroke, epilepsy, Parkinson's disease, head injury, central nervous infection, major depression, or other neurological and psychiatric diseases; acute cardiovascular disease (CVD), cancer, thyroid dysfunction, or other medical illness; auditory or visual disorders; history of alcohol abuse or drug abuse; and history of medical drugs that possibly affect or known to affect cognition.

### Clinical data collection

Demographic variables including ethnicity, age, gender, occupation, educational attainment, and history of smoking and drinking, were collected through a standardized interview. Detailed medical history including duration of diabetes, diabetes complications, current treatment for diabetes, hypertension (including treatment), and CVD, were recorded. Weight and height were used to calculate the body mass index [BMI = weight/height2 (kg/m2)]. Hypertension was defined as a systolic blood pressure of ≥140 mmHg or a diastolic blood pressure of ≥90 mmHg (Chobanian et al., 2003), or indicated by a medical history of antihypertensive drugs. Blood samples were extracted to assess the fasting blood glucose (FBG), glycosylated hemoglobin (HbA1c), c peptide levels after a standard oral glucose tolerance test, thyroid stimulating hormone (TSH), serum creatinine (Scr), blood uric acid, triglyceride (TG), total cholesterol (TC), low-density lipoprotein cholesterol (LDL-c), and high-density lipoprotein cholesterol (HDL-c). Estimated glomerular filtration rate (eGFR) was calculated as follows: [(140-age)^*^weight]^*^(0.85 if female)/Scr^*^72. All parameters were measured in the laboratory of ZhongDa Hospital of Southeast University.

### Measurement of plasma clusterin

Plasma clusterin levels were measured using enzyme-linked immunosorbent assay (ELISA) kits (DCLU00, R&D Systems, Minneapolis, MN, USA) according to the manufacturer's instructions. The minimum detectable concentrations of clusterin was 3.1 ng/ml. Blood samples of all the participants were measured on the same day.

### Neuropsychological tests

All patients underwent a follow-up neuropsychological tests to assess their cognitive function including attention, immediate memory, delayed memory, semantic memory, executive function, visual–spatial ability, and psychomotor speed: Montreal cognitive assessment (MoCA), auditory verbal learning test (AVLT), logic memory test (LMT), complex figure test (CFT), digit span test (DST), trail making test-A (TMT-A), trail making test-B (TMT-B), verbal fluency test (VFT), clock drawing test (CDT). Self-rating depression scale, Hamilton depression rating scale, Hachinski ischemic scale, ADL scale, and CDR scale were also used. An experienced neuropsychiatrist from the Department of Neurology, Affiliated ZhongDa Hospital of Southeast University performed all the tests.

### Genotyping of CLU rs11136000 polymorphism

DNA was extracted from venous blood by using a DNA purification kit (158389, Puregene, GentraSystem, Minneapolis, MN) according to the manufacturer's instruction. Polymerase chain reaction (PCR) was performed to amplify the CLU gene (rs11136000) by using the upstream primer 5′-GTGCCTACCCTTGCTCCA-3′ and the downstream primer 5′-TCACTACAACCTCCGCCTC-3′. PCR was performed in 30 μL reactions containing 20.8 μL of ddH2O, 3.0 μL of 10 × PCR buffer, 2 μL of DNA, 1 μL of primer forward, 1 μL of primer reverse, 2 μL of dNTP, and 0.2 μL of Ex Taq. The PCR conditions were as follows: preliminary denaturation at 96°C for 5 min followed by 30 cycles of denaturation at 96°C for 20 s, annealing at 62°C for 20 s, and extension at 72°C for 30 s, with a final extension step at 72°C for 10 min. The PCR products were electrophoresed on 2.0% agarose gel.

### Statistical analysis

Data were expressed as mean±standard deviation, median (interquartile range), or percentage. Normally distributed variables were analyzed by Student's *t*-test and ANOVA, whereas asymmetrically distributed variables were analyzed using non-parametric Mann−Whitney U and Kruskal−Wallis tests. The Chi-squared test was conducted to compare non-continuous variables and analyzed Hardy-Weinberg equilibrium of the distributions of genotype and allele frequencies. Partial correlation was used to evaluate the relationships between neuropsychological test scores and plasma clusterin. Simple logistic regression model was first performed to select independent factors that increase the selection risk of MCI in T2DM patients. Multivariable regression was subsequently used to investigate the “strongest” factor among the independent risk factors for MCI in T2DM patients. Odds ratios (ORs) and 95% confidence interval (CI) were presented after adjusted for the significant covariates, such as age, education and gender. Statistical analyses were performed using SPSS 20.0 (IBM Corporation). A *p*-value of < 0.05 indicated statistical significance.

## Results

### Demographic, clinical, and cognitive characteristics

Table 1 shows the baseline characteristics of the recruited patients. Among the enrolled T2DM patients, 126 exhibited MCI and 105 showed healthy cognition. Compared with the diabetic patients with healthy cognition, the diabetic patients with MCI were older, attained lower education levels, have been suffering from diabetes for a longer period, and a greater fraction of them were diagnosed with CVD. Patients with MCI displayed a significantly higher level of HbA1c and lower level of HDL-c than the control group. No significant differences between two groups were found regarding gender, BMI, history of smoking and drinking, blood pressure, FBG, C peptide, TG, TC, LDL-c, Scr, blood uric acid, TSH levels, presence of neuropathy, diabetic nephropathy, and treatment with insulin, oral hypoglycemic drugs, and antihypertensive medications (all *p* > 0.05). Compared with the control group, the MCI group showed a significantly higher level of plasma clusterin (104.24 ± 32.01 vs. 115.67 ± 31.59 ug/ml, *p* = 0.007). Neuropsychological test scores were significantly lower in the MCI group than in the non-MCI group (*p* < 0.05).

**Table 1:** **Demographic, clinical and cognitive characteristics of type 2 diabetic patients**.

**Characteristic**	**MCI group (*n* = 126)**	**Control group (*n* = 105)**	***p*-value**
Age (years)^*^	62.56 ± 8.53	58.71 ± 7.97	<0.001
Gender (female/male)	60/66	41/64	0.191
Educational attainment (years)^*^	9.00 (6.00–12.00)	10.00 (9.00–13.00)	<0.001
Ever smoked, n (%)	53 (42.06)	48 (45.71)	0.578
Drinking, n (%)	35 (27.78)	26 (24.76)	0.605
BMI (kg/m^2^)	25.17 ± 3.53	24.99 ± 3.37	0.696
Hypertension, n (%)	82 (65.08)	63 (60.00)	0.427
SBP (mmHg)	138.00 (122.00–148.00)	130.00 (124.00–140.00)	0.208
DBP (mmHg)	80.00 (75.00–90.00)	80.00 (75.00–90.00)	0.654
Diabetes duration (years)^*^	10.67 ± 6.12	8.95 ± 4.99	0.022
HbA1c (%)^*^	9.50 ± 2.15	8.90 ± 2.32	0.042
FBG (mmol/L)	8.20 ± 2.84	7.75 ± 2.67	0.228
Fasting C peptide (ng/mL)	1.96 ± 2.37	1.72 ± 1.06	0.377
C peptide (120 min)	4.93 ± 2.85	4.97 ± 2.93	0.936
TG (mmol/L)	1.67 (1.13–2.44)	1.49 (1.13–2.50)	0.666
TC (mmol/L)	4.92 ± 1.56	4.88 ± 1.24	0.855
LDL- c (mmol/L)	2.91 ± 0.89	2.97 ± 0.87	0.627
HDL- c (mmol/L)^*^	1.18 ± 0.30	1.26 ± 0.24	0.030
Serum creatinine (umol/L)	71.50 ± 21.48	74.47 ± 19.75	0.309
eGFR (mL/min per 1.73 m^2^)	91.67 ± 36.06	93.48 ± 31.48	0.706
Blood uric acid (umol/L)	285.84 ± 93.13	282.62 ± 83.09	0.796
TSH (uul/mL)	2.12 (1.24–3.06)	1.94 (1.33–3.58)	0.757
Coronary heart disease (%)^*^	40 (31.75)	21 (20.00)	0.044
Diabetic nephropathy (%)	34 (26.98)	22 (20.95)	0.287
Neuropathy (%)	26 (20.63)	20 (19.05)	0.764
Clusterin (ug/ml)^*^	115.67 ± 31.59	104.24 ± 32.01	0.007
Use of insulin (%)	86 (68.25)	70 (66.67)	0.798
Oral hypoglycemic drugs (%)	104 (82.54)	82 (78.10)	0.396
Antihypertensive medications (%)	72 (57.14)	47 (44.76)	0.061
**COGNITION TEST LEVELS**			
MoCA^*^	21.00 (17.75–23.00)	27.00 (26.00–28.00)	<0.001
AVLT _IR^*^	15.68 ± 4.46	20.12 ± 4.57	<0.001
AVLT_DR^*^	4.54 ± 2.23	7.09 ± 2.53	<0.001
LMT_IR^*^	5.79 ± 3.94	10.16 ± 4.16	<0.001
LMT_DR^*^	4.68 ± 3.58	8.11 ± 3.74	<0.001
CFT_DR^*^	8.00 (7.00–9.00)	16.00 (14.00–18.00)	<0.001
DST^*^	11.00 (8.00–12.00)	12.00 (11.00–14.00)	<0.001
TMT-A^*^	83.00 (60.00–110.00)	58.00 (48.00–70.50)	<0.001
TMT-B^*^	198.00 (150.00–277.00)	143.00 (111.50–179.50)	<0.001
VFT^*^	15.14 ± 3.94	17.50 ± 4.03	<0.001
CDT^*^	3.00 (2.00–4.00)	4.00 (4.00–4.00)	<0.001

* *Significance, p < 0.05; comparing patients with MCI and those without MCI (controls). Data are presented as n (%), mean ± SD, or median (interquartile range) as appropriate. Student's t test, Mann-Whitney U test, or χ^2^ test was used to test for significant differences. Abbreviations: MCI, mild cognitive impairment; BMI, body mass index; SBP, systolic blood pressure; DBP, diastolic blood pressure; HbA1c, glycosylated hemoglobin; FBG, fasting blood-glucose; TG, triglyceride; TC, total cholesterol; LDL-c, low density lipoprotein cholesterol; HDL-c, high density lipoprotein cholesterol; eGFR, estimated glomerular filtration rate; TSH, thyroid stimulating hormone; MoCA, Montreal Cognitive Assessment; AVLT_IR, auditory verbal learning test, immediately recall; AVLT_DR, auditory verbal learning test, delayed recall; LMT_IR, logic memory test, delayed recall; LMT_DR, logic memory test, delayed recall; CFT_DR, complex figure test, delayed recall; DST, Digit Span Test; TMT-A, Trail Making Test-A; TMT-B, Trail Making Test-B; VFT, Verbal Fluency Test; CDT, Clock Drawing Test*.

### Relationship between cognitive performances and plasma clusterin

Given that significant differences between two groups were observed in terms of neuropsychological test scores and plasma clusterin, partial correlations between neuropsychological test scores and plasma clusterin were conducted. The results showed that plasma clusterin level was negatively correlated with MoCA score and AVLT-delayed recall in the MCI subgroup (*r* = −0.302, *p* = 0.027; and *r* = −0.315, *p* = 0.020, respectively) after adjusting the age, educational attainment, and gender. No significant correlations between LMT_IR, LMT_DR, CFT_DR, DST, TMT-A, TMT-B, VFT, or CDT and plasma clusterin were found (*p* > 0.05; Table 2).

**Table 2:** **Partial correlations of plasma clusterin concentrations with neuropsychological tests in T2DM patients with MCI**.

	**Control variables**	***r***	***p*-value**
MoCA^*^	Age, educational attainment, and gender	−0.302	0.027
AVLT _IR		−0.253	0.065
AVLT_DR^*^		−0.315	0.020
LMT_IR		−0.084	0.547
LMT_DR		−0.079	0.569
CFT_DR		−0.054	0.701
DST		0.129	0.352
TMT-A		0.037	0.793
TMT-B		0.064	0.643
VFT		−0.105	0.451
CDT		−0.046	0.742

* *Significance, p < 0.05. Partial correlation was used. Abbreviations: T2DM, type 2 diabetes mellitus; MCI, mild cognitive impairment; MoCA, Montreal Cognitive Assessment; AVLT_IR, auditory verbal learning test, immediately recall; AVLT_DR, auditory verbal learning test, delayed recall; LMT_IR, logic memory test, delayed recall; LMT_DR, logic memory test, delayed recall; CFT_DR, complex figure test, delayed recall; DST, Digit Span Test; TMT-A, Trail Making Test-A; TMT-B, Trail Making Test-B; VFT, Verbal Fluency Test; CDT, Clock Drawing Test*.

### Distributions of CLU genotype and allele frequencies in MCI and non-MCI groups

Table 3 shows the distributions of CLU genotype and allele frequencies in the two groups. The CLU genotype was consistent with Hardy Weinberg equilibrium in non-MCI group (χ^2^ = 0, *df* = 1, *p* > 0.05) and MCI group (χ^2^ = 0.93, *df* = 1, *p* > 0.05). In addition, no differences were observed between the two groups in terms of distributions of CLU genotype (χ^2^ = 2.709, *df* = 2, *p* = 0.258) and allele (*OR* = 0.713, χ^2^ = 2.038, *p* = 0.153). With CC genotype as reference, the OR of genotype TT was 0.158 (*OR* = 0.158, χ^2^ = 4.113, *p* = 0.043) after adjustment for age, gender, and educational attainment.

**Table 3:** **Distributions of CLU genotype and allele frequencies between groups**.

**CLU genotype**	**MCI, n (%)**	**Non-MCI, n (%)**	**Crude OR (95% CI)**	***p*-value**	**Adjusted OR (95% CI)^b^**	***p*-value**
Overall	126	105				
C	210 (83.33)	164 (78.10)	1.000			
T	42 (16.67)	46 (21.90)	0.713 (0.448–1.136)	0.153		
CC	86 (68.25)	64 (60.95)	1.000			
CT	38 (30.16)	36 (34.29)	0.786 (0.449–1.374)	0.397	0.662 (0.358–1.226)	0.190
TT	2 (1.59)	5 (4.76)	0.298 (0.056–1.584)	0.267^a^	0.158 (0.027–0.941)	0.043^*^
CT+TT	40 (31.75)	41 (39.05)	0.726 (0.422–1.249)	0.247	0.588 (0.323–1.071)	0.083

* Significance, p < 0.05. Data are presented as n (%). The genotype and allele frequencies were compared between the groups with Pearson‘s χ^2^ tests except for the cases labeled with

a , in which the frequency was determined by continuity-corrected Pearson‘s χ^2^ tests.

b *Adjusted for age, educational attainment, gender. Abbreviations: CLU, clusterin; MCI, mild cognitive impairment*.

### Comparison of plasma clusterin and cognitive performances between different CLU genotypes

No significant differences in plasma clusterin levels were observed among the CLU genotypic subgroups (CC, CT, and TT) in either MCI or non-MCI group (*p* > 0.05; Table 4). In addition, no significant differences were identified between genotypic subgroups in terms of neuropsychological test scores in either MCI or control groups (*p* > 0.05; Table 5).

**Table 4:** **The plasma levels of clusterin (ug/ml) in T2DM patients with MCI or normal cognition**.

	**Genotype**			
**Group**	**CC**	**CT**	**TT**	***p*-value**
Non-MCI	104.04 ± 35.96	103.97 ± 24.88	108.87 ± 28.78	0.948
MCI	114.66 ± 31.91	117.23 ± 31.87	129.72 ± 5.02	0.753
Total	110.13 ± 33.99	110.78 ± 29.26	114.82 ± 25.69	0.928

*Data are presented as mean ± SD. One-way ANOVA for the comparison of the plasma level of clusterin among different genotypes. Abbreviations: T2DM, type 2 diabetes mellitus; MCI, mild cognitive impairment*.

**Table 5:** **Comparison of cognitive performance according to genotypes of CLU gene rs11136000 polymorphism**.

**Cognitive performance**	**MCI group (*N* = 126)**				**Control group (*N* = 105)**			
	**CC (*n* = 86)**	**CT (*n* = 38)**	**TT (*n* = 2)**	***p*-value**	**CC (*n* = 64)**	**CT (*n* = 36)**	**TT (*n* = 5)**	***p*-value**
MoCA	21.00 (17.00–22.25)	22.00 (18.50–24.00)	(21.00–21.50)	0.108	27.00 (26.00–28.00)	27.00 (26.00–28.00)	26.00 (25.50–26.50)	0.121
AVLT _IR	15.69 ± 4.63	16.08 ± 4.12	10.50 ± 0.71.	0.238	19.39 ± 3.98	20.92 ± 5.09	22.40 ± 4.57	0.206
AVLT_DR	4.24 ± 2.25	5.21 ± 2.13	3.00 ± 1.41	0.142	6.00 (5.00–9.00)	7.00 (5.75–10.00)	8.00 (6.00–10.00)	0.290
LMT_IR	5.56 ± 3.82	6.29 ± 4.19	5.00 ± 5.66	0.736	10.84 ± 3.80	9.12 ± 4.72	9.60 ± 3.72	0.236
LMT_DR	4.00 (1.00–7.50)	5.50 (1.00–8.75)	(1.00–4.50)	0.813	8.61 ± 3.47	7.58 ± 4.30	6.40 ± 2.30	0.310
CFT_DR	8.02 ± 1.79	8.63 ± 1.95	7.50 ± 2.12	0.380	15.66 ± 2.83	16.04 ± 2.93	15.60 ± 3.05	0.858
DST	11.00 (8.00–12.00)	10.00 (8.00–11.00)	10.00 (10.00–10.00)	0.465	12.38 ± 2.00	11.83 ± 1.73	12.20 ± 2.17	0.402
TMT-A	83.00 (60.75–107.25)	82.50 (56.00–114.75)	(60.00–106.00)	0.923	61.73 ± 18.29	60.28 ± 21.18	80.00 ± 19.24	0.105
TMT-B	195.00 (150.00–254.75)	215.00 (147.00–333.75)	(198.00–205.50)	0.601	145.27 ± 46.59	156.44 ± 61.95	195.80 ± 58.55	0.099
VFT	14.78 ± 3.84	15.92 ± 3.94	16.00 ± 2.83	0.371	17.59 ± 3.77	17.47 ± 4.47	16.60 ± 4.83	0.869
CDT	3.00 (2.00–4.00)	3.00 (2.00–4.00)	4.00 (4.00–4.00)	0.310	4.00 (4.00–4.00)	4.00 (4.00–4.00)	4.00 (3.50–4.00)	0.921

*Data are presented as mean ± SD, or median (interquartile range) as appropriate. Analysis of variance (ANOVA) for comparison of normally distributed quantitative variables between genotypic subgroups in MCI group and control group. Kruskal-Wallis test for comparison of asymmetrically distributed quantitative variables between genotypic subgroups in MCI group and control group. Abbreviations: CLU, clusterin; MCI, mild cognitive impairment; MoCA, Montreal Cognitive Assessment; AVLT_IR, auditory verbal learning test, immediately recall; AVLT_DR, auditory verbal learning test, delayed recall; LMT_IR, logic memory test, delayed recall; LMT_DR, logic memory test, delayed recall; CFT_DR, complex figure test, delayed recall; DST, Digit Span Test; TMT-A, Trail Making Test-A; TMT-B, Trail Making Test-B; VFT, Verbal Fluency Test; CDT, Clock Drawing Test*.

### Logistic regression models

To investigate the risk factors of MCI in T2DM, we conducted univariate logistic regressions to select the so-called independent variables for multivariable regression model. The independent variables used in the simple logistic regression model include age, gender, educational attainment, history of smoking and drinking, duration of diabetes, FBG, HbA1c, fasting C peptide, C peptide (120 min), TG, TC, LDL-c, HDL-c, serum creatinine, eGFR, blood uric acid, TSH, SBP, DBP, prevalence of hypertension, CVD, diabetic nephropathy, neuropathy, plasma clusterin level and CLU genotype. The results showed that the variables associated with MCI in T2DM diabetes include old age, low education levels, long duration of diabetes, low HDL-c level, CVD history, and high HbA1c and clusterin levels (Table 6).

**Table 6:** **Assessment results of the risk of having MCI in a simple logistic regression model in T2DM patients**.

	***B***	***SE***	***p*-value**
Age (years)^*^	−0.056	0.017	0.001
Gender (female/male)	−0.350	0.268	0.192
Educational attainment (years)^*^	0.185	0.040	<0.001
Ever smoked, n (%)	0.148	0.266	0.578
Drinking, n (%)	−0.156	0.301	0.605
BMI (kg/m^2^)	−0.015	0.038	0.694
Hypertension, n (%)	−0.204	0.107	0.055
SBP (mmHg)	−0.013	0.008	0.124
DBP (mmHg)	−0.012	0.013	0.347
Diabetes duration (years)^*^	−0.055	0.024	0.023
HbA1c (%)^*^	−0.123	0.061	0.044
FBG (mmol/L)	−0.061	0.050	0.228
Fasting C peptide (ng/mL)	−0.081	0.097	0.400
C peptide (120 min)	0.004	0.055	0.935
TG (mmol/L)	−0.036	0.077	0.635
TC (mmol/L)	−0.017	0.094	0.854
LDL-c (mmol/L)	0.074	0.152	0.625
HDL-c (mmol/L)^*^	1.053	0.492	0.032
Serum creatinine (umol/L)	0.007	0.007	0.308
eGFR (mL/min per 1.73 m^2^)	0.002	0.004	0.705
Blood uric acid (umol/L)	0.000	0.002	0.795
TSH (uul/mL)	−0.034	0.075	0.655
Coronary heart disease (%)^*^	−0.621	0.310	0.045
Diabetic nephropathy (%)	−0.332	0.313	0.288
Neuropathy (%)	−0.100	0.332	0.716
Clusterin (ug/ml)^*^	−0.012	0.004	0.008
CLU genotype	0.354	0.244	0.147

* *Significance, p < 0.05. Abbreviations: MCI, mild cognitive impairment; T2DM, type 2 diabetes mellitus; BMI, body mass index; SBP, systolic blood pressure; DBP, diastolic blood pressure; HbA1c, glycosylated hemoglobin; FBG, fasting blood-glucose; TG, triglyceride; TC, total cholesterol; LDL-c, low density lipoprotein cholesterol; HDL-c, high density lipoprotein cholesterol; eGFR, estimated glomerular filtration rate; TSH, thyroid stimulating hormone; CLU, clusterin*.

Multivariable regression revealed that educational attainment, duration of diabetes, and HDL-c and clusterin levels are associated with MCI in T2DM patients (Table 7).

**Table 7:** **Assessment results of the risk of having MCI in a multivariable logistic regression model in patients with T2DM**.

	***B***	***SE***	***p*-value**
Educational attainment (years)^*^	0.189	0.041	<0.001
Diabetes duration (years)^*^	−0.059	0.026	0.024
HDL- c (mmol/L)^*^	1.189	0.545	0.029
Clusterin (ug/ml)^*^	−0.011	0.005	0.019

* *Significance, p < 0.05. Abbreviations: MCI, mild cognitive impairment; T2DM, type 2 diabetes mellitus; HDL-c, high density lipoprotein cholesterol*.

## Discussion

Our study suggested that plasma clusterin concentration increased and was negatively correlated with memory performance in T2DM patients with MCI. TT genotype carriers demonstrated a reduced MCI risk compared with CC genotype carriers after adjustment for age, educational attainment, and gender. High clusterin level, low HDL-c concentration, long duration of diabetes, and low educational attainment are associated with MCI in T2DM patients. No significant differences in plasma clusterin levels or neuropsychological test scores were observed among CLU genotypes either in MCI or control groups.

Consistent with previous results (Thambisetty et al., 2012; Mullan et al., 2013; Jongbloed et al., 2015), plasma clusterin level was higher in MCI patients than in cognitively healthy group. Similar results were observed in studies that compared AD patients with control participants (Schrijvers et al., 2011; Xing et al., 2012; Mullan et al., 2013). However, in contrast to our result, some studies have found that plasma clusterin levels were not increased in patients with presymptomatic AD or in individuals who later developed AD or in AD patients, compared with individuals who remained cognitively healthy (IJsselstijn et al., 2011; Silajdzic et al., 2012; Jongbloed et al., 2015). This disparity may be attributed to the difference in study populations (MCI or AD). One plausible explanation is that in the early stage, a glial cell and Aβ driven pro inflammatory response extends from central (AD-affected areas) to the periphery, leading to the clusterin upregulation in AD affected areas, as same as in periphery (May et al., 1990; Lidstrom et al., 1998). In the subsequent stage, the inflammatory response, which involves microglial activation and cytokine release, subsides (Eikelenboom et al., 2012), making plasma clusterin levels to drop to control levels in AD. Another possible interpretation is that increased plasma clusterin level is related to a reduced rate of atrophy as a protective factor in cognitive impairment patients (Malkki, 2014). However, in established AD subjects, reduced plasma clusterin level may reflect exhaustion of such protective mechanisms caused by genetic and/or infaust environmental effects (Thambisetty et al., 2012). Thus, whether plasma clusterin serves as a deleterious substance or reflects a neuroprotective response during progress of cognitive impairment warrants further research.

Our results showed that plasma clusterin was correlated with MoCA scores in MCI patients, similar to findings of Meng et al. (2015) and Schrijvers et al. (2011), who studied MCI patients and AD patients, respectively. MoCA is a highly sensitive tool to distinguish MCI from cognitively healthy T2DM patients (Alagiakrishnan et al., 2013). In addition, plasma clusterin in MCI patients was associated with AVLT-delayed recall, which evaluates episodic memory function. Impairment in episodic memory function is the earliest change in presymptomatic stages of AD (Grober et al., 2008). Our results were consistent with the finding that plasma clusterin reflects cognitive function in MCI patients (Meng et al., 2015) and predicts increased entorhinal cortex amyloid burden, which is important in memory formation (Thambisetty et al., 2010). Several characteristic properties of clusterin make it related with the pathology of cognitive impairment. First, clusterin inhibits amyloid formation by binding Aβ and preventing their aggregation (Desikan et al., 2014). Second, clusterin is involved in the clearance of Aβ by enhancing endocytosis in glial cells (Tanzi et al., 2004; Wang et al., 2006) and by assisting in Aβ transport through the blood-brain barrier (Zlokovic et al., 1994; Zlokovic, 2004). Third, clusterin may increase cholesterol transport in conjunction with ApoE in blood vessels (Gelissen et al., 1998). Fourth, clusterin can inhibit the activation of complement (Calero et al., 1999). What's more, being a chaperone, clusterin can bind to Smad2/3 proteins to enhance neuroprotective TGFβ signaling (Lee et al., 2008). In addition, clusterin can be conveyed into the cytoplasm to bind to Bax protein and suppress neuronal apoptosis (Nuutinen et al., 2009). These lines of evidence demonstrated that neurodegenerative changes during cognitive impairment may increase clusterin expression (Nuutinen et al., 2009), suggesting the beneficial role of clusterin in AD pathology.

Compared with the studies indicating that the rs1113600 minor T allele occurs less frequently in AD patients than in cognitively healthy control patients and that CLU rs11136000 CC genotype is a the risk factor for AD (Bertram et al., 2007; Carrasquillo et al., 2015), our study found no difference in the distribution of genotype and allele frequencies of CLU rs11136000 in T2DM patients with MCI and controls, possibly because of the low minor allele frequency in Chinese and the small sample size. However, after adjustment for age, educational attainment, and gender, our data showed that compared with the CC genotype, the TT genotype is associated with reduced risk for T2DM-related MCI, validating the finding that protective association existed between T allele or TT genotype and AD (Harold et al., 2009; Lambert et al., 2009). The following mechanisms may explain a high risk of MCI happened in CC carriers compared with CT/TT genotype carriers. Compared to TT/CT genotype, enhanced effects on midline default mode network (DMN) in CC genotype was found in MCI subjects (Bai et al., 2016). Midline DMN regions may have an information-bridging role to task-positive networks (Elton and Gao, 2015) which is activated when the brain processes externally presented information (Kim et al., 2010). What's more, CC genotype may initially influence restingstate networks integrity and then lead to cognitive impairment (Bai et al., 2016). On the other hand, CC genotype was associated with increased alpha3 absolute power and changes of alpha1 activity in topographical distribution. The increased synchronization of upper alpha activity are considered to be associated with the changes in cortical networks and hippocampal (Ponomareva et al., 2013). In addition, CC genotype carriers may be associated with beta amyloid deposition than TT genotype carriers (Tan et al., 2016). Despite above-mentioned explanations, the underlying mechanisms are not fully understood.

In this study, rs11136000 genotypes exerted no significant effect on plasma clusterin level in the MCI group, in the control group, or in both, consistent with previous finding showed that no effect of polymorphisms in rs11136000 on plasma clusterin among AD, MCI, and control subjects analyzed together in white Europeans from United Kingdom, France, Italy, Finland, Poland, and Greece (Thambisetty et al., 2010). By contrast, in the study conducted in Germany, an increased level of plasma clusterin in rs11136000 TT carriers than CC carriers in cognitive healthy individuals, while no difference was observed in AD patients (Schurmann et al., 2011). In Northern Ireland population, researchers found lower plasma clusterin level in TT homozygotes in subjects with healthy-cognition (Mullan et al., 2013), while genotype at this SNP showed no influence on plasma clusterin when control, MCI and AD individuals were analyzed together. The following factors may explain the conflicting findings. Disease-related increases of plasma clusterin covered the effect of genotype on clusterin protein, so change of plasma clusterin level only observed in the control group and not in groups analyzed together. Another interpretation is that the association between plasma clusterin and AD pathological processes is probably independent of genetic variation (Thambisetty et al., 2010) or rs11136000 may not be the clusterin SNP that induces expression or functional changes in clusterin protein. Thus, larger sample size studies combining analysis of clusterin protein concentration and CLU gene variation are needed to validate this finding. In addition, our data suggested that no significant relationship exists between rs11136000 polymorphism of the CLU gene and the cognitive performance domains, such as executive function and memory performance. In contrast to our results, a previous study has found high and low memory scores in individuals with protective and risk alleles of CLU rs11136000 SNP, respectively (Thambisetty et al., 2013; Pedraza et al., 2014). Several conditions may explain this difference. First, the participants in the current study are Chinese Han patients, which are different from the Caucasian population (Thambisetty et al., 2013; Pedraza et al., 2014). Second, cognitive impairment during AD progression is complicated and affected by several genes (Cox et al., 2014); rs11136000 polymorphism may have only exerted a slight influence. Third, mutual effects of gene and environment should have been considered (Rajan et al., 2014). Despite all these factors, we believe that an underlying association exists between memory decline and CLU gene, considering that a recent study has found that neural hyperactivation occurred in the frontal cortex, posterior cingulate cortex, and hippocampus during a working memory task in healthy young adults carry AD risk variant in CLU (Lancaster et al., 2011).

In our study, multivariable logistic regression model shows that educational attainment, duration of diabetes, low HDL-C, as well as clusterin are associated with MCI in diabetic patients. Except that people with higher education have less chance to develop MCI, study also suggested that high education was a protective factor for the conversion from MCI to AD (Tervo et al., 2004). However, the underlying mechanism needs further investigation. There is a growing awareness that patients with longer diabetes duration had severe condition of oxidative stress (Polidori et al., 2000). Oxidative stress plays an important role in the development of atherosclerosis as well as cognitive impairment in T2DM (Mackness et al., 1997; Ahmad, 2013). Scholars have found that clusterin was accumulated in the artery wall to protect against the oxidative stress (Mackness et al., 1997). For the same reason, elevated plasma clusterin may also represent a protective mechanism against cognitive impairment in response to oxidative stress that caused by longer diabetes duration (Thambisetty et al., 2012). In plasma, an important source of clusterin is associated with HDL particles (Calero et al., 1999). Several studies in different populations have suggested that high HDL-c levels were associated with a reduced risk for AD (Launer et al., 2001; Reitz et al., 2004; Vollbach et al., 2005). Another study has found a strong positive correlation between clusterin levels in HDL and insulin sensitivity which is important during pathology of diabetes-associated MCI (Hoofnagle et al., 2010; Roberts et al., 2014). Thus, we speculate that increased plasma clusterin possibly plays a protective role in the early stage of AD. It is worth noting that we identified no different prevalence of MCI between man and women. Also, gender is not the independent factor that influence the likely of having MCI in T2DM patients. Other studies have found a similar result although different methods of diagnostic criteria for MCI were used (Hanninen et al., 2002; Solfrizzi et al., 2004). On the contrary, either a higher prevalence of MCI in men than women (Ganguli et al., 2004; Petersen et al., 2010) or a higher prevalence of MCI in women compared with men were observed in different population (Larrieu et al., 2002; Di Carlo et al., 2007). Except for the difference of ethnicity, we consider the diabetic status of subjects in this study increase the prevalence of MCI that narrowed the gap between men and women.

This study provides significant insights into plasma clusterin and CLU rs11136000 polymorphism underlying MCI in T2DM patients. However, certain limitations should not be ignored. First, the sample size was relatively small and thus, results of this study should be interpreted carefully. Second, the study was not a longitudinal prospective research; AD patients were not included in this study and thus we could not clarify the clusterin variation during AD progression; therefore, we cannot determine whether plasma clusterin is a preclinical marker of AD. Third, magnetic resonance imaging was not performed in this study, so brain pathologic change of cognitive impairment under the state of T2DM is unclear.

## Conclusion

In summary, our data showed that plasma clusterin is increased in T2DM patients with MCI, compared with cognitively healthy controls. CLU rs11136000 TT genotype is associated with a reduced MCI risk compared with the CC genotype after adjustment for age, educational attainment, and gender. Elevated plasma clusterin levels may reflect a neuroprotective response in T2DM patients with MCI. Further studies need to be conducted to determine whether clusterin can be used as an underlying early marker of diabetes-associated MCI; in addition, studies with a larger sample-size should be performed to investigate the association between rs11136000 polymorphism and MCI in T2DM. As an option, interventions that stimulate clusterin expression probably result in a beneficial effect for subjects with cognitive decline.

## Author contributions

SW contributed to the idea and revised the manuscript. RC carried out the design, conduct of the study and wrote the manuscript. JH, JS carried out the data collection. RH and ST participated in the data analysis. YS, XD, and WX helped data interpretation. All authors read and approved the final manuscript.

## Funding

This work was partially supported by the National Natural Science Foundation of China (No.81570732, SW and No. 81370921, SW).

### Conflict of interest statement

The authors declare that the research was conducted in the absence of any commercial or financial relationships that could be construed as a potential conflict of interest.

## Footnotes

^1^Available: http://www.diabetesatlas.org/ [Accessed].
